# An Assessment of Blood Vessel Remodeling of Nanofibrous Poly(ε-Caprolactone) Vascular Grafts in a Rat Animal Model

**DOI:** 10.3390/jfb14020088

**Published:** 2023-02-03

**Authors:** Jana Horakova, Tereza Blassova, Zbynek Tonar, Connor McCarthy, Katerina Strnadova, David Lukas, Petr Mikes, Patrick Bowen, Roger Guillory, Megan Frost, Jeremy Goldman

**Affiliations:** 1 Department of Nonwoven and Nanofibrous Materials, Technical University of Liberec, Studentska 1402/2, 461 17 Liberec, Czech Republic; 2 Department of Histology and Embryology and Biomedical Centre, Faculty of Medicine in Pilsen, Charles University, Karlovarska 48, 301 66 Pilsen, Czech Republic; 3 Department of Biomedical Engineering, Michigan Technological University, 1400 Townsend Drive, Houghton, MI 49931, USA; 4 Department of Chemistry-Bioengineering, Faculty of Science, Humanities and Education, Technical University of Liberec, Studentska 1402/2, 461 17 Liberec, Czech Republic; 5 Department of Physics, Faculty of Science, Humanities and Education, Technical University of Liberec, Studentska 1402/2, 461 17 Liberec, Czech Republic; 6 Department of Materials Science and Engineering, Michigan Technological University, 1400 Townsend Drive, Houghton, MI 49931, USA

**Keywords:** vascular graft, electrospinning, polycaprolactone, vascular remodeling, histological evaluation, stereology

## Abstract

The development of an ideal vascular prosthesis represents an important challenge in terms of the treatment of cardiovascular diseases with respect to which new materials are being considered that have produced promising results following testing in animal models. This study focuses on nanofibrous polycaprolactone-based grafts assessed by means of histological techniques 10 days and 6 months following suturing as a replacement for the rat aorta. A novel stereological approach for the assessment of cellular distribution within the graft thickness was developed. The cellularization of the thickness of the graft was found to be homogeneous after 10 days and to have changed after 6 months, at which time the majority of cells was discovered in the inner layer where the regeneration of the vessel wall was found to have occurred. Six months following implantation, the endothelialization of the graft lumen was complete, and no *vasa vasorum* were found to be present. Newly formed tissue resembling native elastic arteries with concentric layers composed of smooth muscle cells, collagen, and elastin was found in the implanted polycaprolactone-based grafts. Moreover, the inner layer of the graft was seen to have developed structural similarities to the regular aortic wall. The grafts appeared to be well tolerated, and no severe adverse reaction was recorded with the exception of one case of cartilaginous metaplasia close to the junctional suture.

## 1. Introduction

Cardiovascular diseases (CVDs) constitute the most common cause of death globally. According to the World Health Organization, more people die annually from CVDs than from any other cause. A large number of patients suffer from vascular damage, thus resulting in the need for bypass surgery. Since limitations remain concerning the replacement of small diameter vascular grafts, the need and demand for the development of more suitable grafts are increasing day by day. While the materials currently employed are commercially fabricated from inert polymers such as expanded polytetrafluorethylene (ePTFE) or polyethylene terephtalate, known as Dacron, research is increasingly being focused on biodegradable materials, since they have been found to possess several advantages over their inert counterparts. Biodegradable polyester poly(*ε*-caprolactone) (PCL) has been reported as a potential candidate for the production of vascular prostheses [1,2,3,4]. A comparison by Pektok et al. of the healing characteristics of vascular grafts made from electrospun PCL with an average fiber diameter of 1.9 μm and commercially available ePTFE following implantation in rats determined that the electrospun PCL conduit exhibited more rapid endothelialization and better cellular infiltration accompanied by neovascularization 6 months following implantation than did the ePTFE [5]. However, while PCL evinces an intrinsically slow degradation rate accompanied by the desirable mechanical properties and general biocompatibility [6], de Valence et al. [7] and Wu et al. [8] have reported the insufficient regeneration of the vascular wall as well as graft calcification.

It is possible to electrospin polycaprolactone into various morphologies so as to form nano- as well as microfibrous structures by varying the solvent system and electrospinning conditions. A study by Wang et al. determined that vascular grafts composed of thicker fibers with a diameter of 5–6 µm proved more successful upon implantation in terms of vessel wall regeneration than did a prosthesis composed of thinner fibers with a diameter of less than 1 µm [9]. Conversely, other studies devoted to the thrombogenicity of electrospun materials have indicated a preference for grafts with smaller fiber diameters (˂1 µm) with respect to lower levels of platelet adhesion and the activation of a coagulation cascade [10]. It is difficult to compare the advantages and disadvantages of nano- and microfibrous materials since the relevant studies have been based on a variety of investigation approaches (in vitro vs. in vivo studies, the utilization of various animal models and blood sources etc.).

In addition to fiber diameter, molecular weight of the used polymer could have a significant impact on final material properties such as mechanical behavior, degradation rate, etc. Table 1 summarizes studies of electrospun polycaprolactone grafts implanted in rat animal models. All the studies are based on polycaprolactone with a molecular weight of 80,000 g/mol. Some of the studies point out the slow rate of biodegradation of the polymer [11]. Therefore, utilization of lower molecular weight polycaprolactone could accelerate the degradation rate, which could enable proper regeneration of the natural vessel wall. Lower molecular weight polymer possesses weaker mechanical properties, which could be further optimized by the thickness of the material. Details of the material properties of electrospun polycaprolactone grafts can be found in our previous study [12].

Following the implantation of a biodegradable material, the body initiates the healing response. Ideally, the scaffold structure and composition should be capable of promoting the healing of injured or damaged tissue. In this case, the scaffold material serves as a temporary support that initiates the self-renewal of the tissue. Following implantation in vivo, the graft provokes an in vivo response known as graft healing; the proposed remodeling process has been described by de Valence et al. [7] and Wang et al. [9]. Macrophages make up the first cells to infiltrate the graft. The inner surface of the graft is endothelialized within a number of months depending on the animal model. Smooth muscle cells penetrate the graft, thus ensuring the physiological function of the implanted graft. Subsequently, *vasa vasorum* develop and nourish the newly created vessel in the adventitial site. Certain adverse effects such as calcification or a foreign body response may be observed [7,9].

A histological assessment of vascular grafts is usually conducted via the evaluation of the endothelial coverage of the lumen, the infiltration of smooth muscle cells, and the immunological reaction, all of which constitute common vessel remodeling features. The various studies devoted to electrospun biodegradable polyesters following implantation as vascular grafts in rat animal models and the histological evaluations thereof are summarized in Table 1. Interestingly, none of the studies cited below employed stereology for the assessment of graft remodeling, in contrast to other studies on the in vivo remodeling of tissue-engineered grafts and scaffolds concerning, e.g., bone research [13,14].

**Table 1:** List of in vivo studies of electrospun polycaprolactone-based small diameter vascular grafts tested in rats.

Study	Material	Assessed Timepoints	Histological Evaluation
Pektok et al. [5]	Electrospun PCL, ePTFE	3, 6, 12, 18, 24 weeks	Cellular infiltration, endothelialization, regeneration of the vessel wall (collagen, elastin), immune response (FBGCs, macrophages), calcification
Nottelet et al. [15]	Electrospun PCL	3, 6, 12 weeks	Cellular infiltration, endothelialization
de Valence et al. [7]	Electrospun PCL	1.5, 3, 6, 12, 18 months	% cell invasion from the adventitia, endothelialization + *vasa vasorum* formation, immune response (FBGCs), regeneration of the vessel wall (smooth muscle cells, collagen, elastin), calcification
de Valence et al. [16]	Electrospun PCL—bi-layered grafts (no barrier, inside/outside/only barrier)	3, 12 weeks	% cell invasion from the adventitia, endothelialization + *vasa vasorum* formation, intimal hyperplasia
de Valence et al. [17]	Electrospun PCL—plasma treated	3 weeks	Cell penetration, cell density
Wang et al. [9]	Electrospun PCL—thicker fibers/thinner fibers	7, 14, 28, 100 days	Cellular infiltration, endothelialization, regeneration of the vessel wall (smooth muscle cells, collagen, elastin), immune response (macrophages)
Tille et al. [18]	Electrospun PCL modified with paclitaxel/dexamethasone	1, 3, 12 weeks	Cellular infiltration, regeneration of the vessel wall (collagen), immune response (macrophages), calcification
Yang et al. [19]	Poly(glycerol sebacate) core + electrospun PCL	3, 12 months	Cellularization, endothelialization + *vasa vasorum* formation, regeneration of the vessel wall (smooth muscle cells, collagen, elastin), immune response (macrophages), calcification
Pan et al. [20]	Co-electrospun PCL/polydioxanone	1, 3 months	Endothelialization, regeneration of the vessel wall (smooth muscle cells, collagen, elastin, glycosaminoglycans), calcification
Wang et al. [21]	Electrospun PCL modified with resveratrol	2, 4 weeks	Cellular infiltration, endothelialization, regeneration of the vessel wall (smooth muscle cells), immune response (macrophages)
Wang et al. [22]	Electrospun PCL modified with proteins (VEGF, HGFI)	1 month	Cell density, endothelialization, endothelialization + *vasa vasorum* formation, regeneration of the vessel wall (smooth muscle cells, collagen, elastin, glycosaminoglycans), immune response (macrophages)
Li et al. [23]	Electrospun PCL—bi-layered graft	3, 18 months	Cellularization, endothelialization + *vasa vasorum* formation, regeneration of the vessel wall (smooth muscle cells, collagen, elastin, glycosaminoglycans), calcification
Wu et al. [8]	Electrospun PCL	12 months	endothelialization, regeneration of the vessel wall (smooth muscle cells, collagen, elastin), immune response (macrophages), calcification
Dokuchaeva et al. [11]	Electrospun PCL	10, 30, 60, 90 days	Cellularization, calcification

Blood vessel remodeling leads to the structure mimicking the native vessel wall, which is composed of three distinct layers. Consequently, our approach was based on the evaluation of cellular infiltration into different parts of the graft. The whole of the thickness of the graft was measured and divided into three regions—abluminal, middle, and adluminal. Cell colonization may be preferred either from the adventitial site from adjacent tissue or from the luminal site (cells from the bloodstream) at the beginning of the healing process. It is believed that three layers are formed following regeneration. The studies listed above investigated cell density or cellularization of implanted grafts in the whole thickness of the material. Stereological assessment based on the division of the graft into three layers enables localization of the cells into specific regions and could be helpful in quantification of regeneration in terms of cell distribution within these virtual layers.

The aim of this article is to submit a proposal for the assessment of the healing characteristics of an implanted prosthesis based on a detailed evaluation of nanofibrous PCL vascular grafts in vivo in a rat animal model. The study was designed so as to provide proof of a low molecular weight nanofibrous PCL vascular graft concept that has not yet been tested in vivo. Histological processing and a stereological evaluation were employed so as to describe the regeneration of a blood vessel prosthesis in an experimental animal model.

## 2. Materials and Methods

### 2.1. Vascular Graft Preparation

Vascular grafts (inner diameter 1.65 mm, 2 cm in length) produced from poly(*ε*-caprolactone) (PCL, Sigma Aldrich, M_n_ 45,000) were obtained via the electrospinning of a 16% (*w*/*w*) solution of PCL dissolved in chloroform/ethanol/acetic acid (8:1:1 v:v:v). The solution was charged at 20 kV and ejected through a 22-gauge needle (Beckton, Dickinson & Co; Franklin Lakes, NJ, USA) at a constant rate of 2.54 mL h^−1^. The fibers were collected on a rotating tubular 304 stainless steel mandrel at a rotational speed of 250 rpm. The needle tip was placed 15 cm above the mandrel. The tubes were removed from the collector following electrospinning and placed under vacuum conditions for 2 h so as to allow for the evaporation of any residual solvent. The samples were sterilized via immersion in 70% ethanol for 10 min prior to implantation.

### 2.2. Morphological Characterization of the Vascular Graft

The fiber morphology was evaluated by means of field emission scanning electron microscopy (FESEM) on a Hitachi S-4700 (5 kV accelerating voltage; 10 μA beam current; Tokyo, Japan). The samples were sputter coated with platinum/palladium on a Hummer 6.2 sputter coater (Anatech Ltd.; Denver, CO, USA) to a thickness of 5 nm and observed at low and high magnification. The fiber diameter was measured using NIH Image J software (*n* = 100).

### 2.3. In Vivo Implantation

The in vivo experimental protocol was approved by the Michigan Technological University Institutional Animal Care and Use Committee (IACUC) board (Project identification code [380482-43] Vascular Materials). NIH guidelines for the care and use of laboratory animals (NIH Publication #85-23 Rev. 1985) were fully observed. Seven Sprague Dawley rats received an abdominal aorta replacement graft with an internal diameter of 1.65 mm. The rats were anesthetized via the inhalation of 2.1% isoflurane in oxygen. A midline laparotomy incision was then performed so as to isolate the abdominal aorta. Side branches from a 10 mm long segment of the artery between the renal and femoral bifurcations were tied off so as to prevent collateral blood flow from within the segment. The proximal and distal ends of the segment were clamped and the artery severed near to the midpoint of the 10 mm segment. A microdose (~10 μL) of 200 units/mL heparin was administered to the exposed ends of the artery. The polymeric grafts were sutured to the exposed ends of the artery using an end-to-end anastomotic technique with 10-0 nylon sutures (15−20 stitches at each end). After suturing both ends of the graft to the native vessel, blood flow was re-established, and the pulsation of the distal artery was observed for the demonstration of blood flow. The abdomen was closed using sutures for the muscle and staples for the skin. The rats recovered from the anesthesia and were provided with food and water ad libitum. No anticoagulation treatment was administered to the rats.

After 10 days (*n* = 3) and 6 months (*n* = 4), the rats were sacrificed by increasing the inhaled isoflurane concentration from 2.1 to 5.0% and then puncturing the diaphragm followed by the removal of the heart after the rats had been deeply anesthetized. The graft was collected with the host distal and proximal ends of the artery. The samples were embedded in freezing medium (Neg 50; Thermo Scientific; Waltham, MA, USA), snap frozen in liquid nitrogen, and stored at −80 °C prior to cryo-sectioning.

### 2.4. Histological Processing

**Figure 1:**
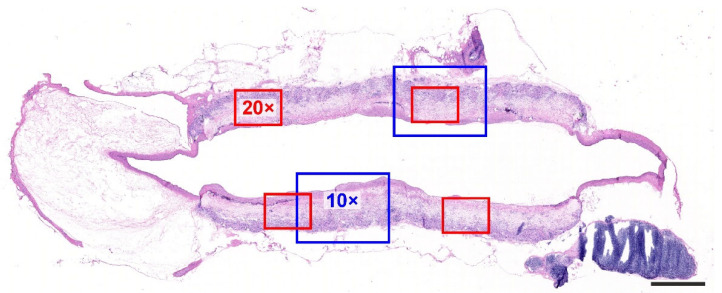
Overall view of the graft attached to the proximal and distal aortic segments, hematoxylin–eosin stain. The proportionally sized blue squares simulate the magnification of the 10× objective, and the size of the red squares relates to the 20× objective. Scale bar = 1 mm.

The early stage of the engraftment of the vascular grafts was analyzed 10 days (*n* = 3), and the long-term response was assessed 6 months (*n* = 4) following implantation. The tissue blocks were cut so as to provide approx. 10 µm-thick histological sections with a section plane parallel to the long axis of the aorta (Figure 1).

The sections were stained using a variety of general and specialized histological (Table 2) as well as immunohistochemical reaction dyes (Table 3) in order to enable the characterization of the cellular populations of the grafts. The visualization of the immunohistochemical reaction was based on diaminobenzidine (DAB+, Liquid; DakoCytomation, Glostrup, Denmark). The immunohistochemical sections were then counterstained with Gill’s hematoxylin. All the sections were dehydrated in graded ethanol solutions and mounted with a xylene-soluble medium.

Stained samples were evaluated qualitatively in order to describe the vessel remodeling in two investigated time points. No statistical analysis was performed due to the low number of tested samples.

### 2.5. Quantification of Cellular Density within the Thickness of the Graft

Cellular infiltration was assessed via the hematoxylin staining of the cell nuclei. All the quantitative estimates were conducted employing well-established stereological methods [28] available in Ellipse and uDis software (ViDiTo, Košice, Slovakia). The quantitative parameters are listed and explained in Table 4. The microscope objective and magnification used in the quantitative assessment of each of the parameters were set at the lowest levels that permitted the exact and unambiguous identification of the counting events with respect to the histological staining methods. Bright field and polarizing microscopy were performed using an Olympus CX51 microscope. The sampled image fields were captured applying a resolution of 1616 × 1912 pixels.

With respect to the quantification of cell density within the graft wall, a cross-section of the graft was evaluated employing stereological techniques. The inner (adluminal) and outer (abluminal) linear border profiles were highlighted in each micrograph using the Ellipse software Multiline tool. Whereas the inner border was always clearly visible, the outer border sometimes also contained connective tissue with adjacent blood vessels that did not belong to the graft but were harvested together with the graft. The outer graft border was therefore defined as the most external continuous part of the graft. The thickness of the graft was estimated using the Ellipse software LocalizeInWall2 module [29] as the mean distance between the points of the inner and outer borders. The nuclei profiles of all the cells between the borders of the grafts were selected using the Ellipse software Gold—Detection plugin [30]. Following the visual control, manual correction in areas of overlapping nuclei profiles was conducted using the Ellipse software Point tool; the nuclei outside the graft area were not considered. The quantity of marked nuclei per section area profile *Q_A_* was assessed using the LocalizeinWall2 module of the same software, as the ratio *Q_A_*= *Q*/*A* (mm^−2^)*,* where *Q* is the number of nuclei profiles counted, and *A* is the estimated reference area of the section through the graft. In order to assess the relative distribution of the nuclei profiles within the graft in more detail, three virtual sublayers (inner, middle, and outer sublayers) were arbitrarily defined within the graft (Figure 3A), each of which had an equal thickness comprising exactly one third of the local graft thickness while respecting all the irregularities of the local graft borders. The inner, middle, and outer sublayers did not consist of the actual morphological layers of the graft but rather ideal thirds that were used for the assessment of the homogeneity of the distribution of the nuclei profiles across the whole thickness of the graft. Finally, the numbers of nuclei profiles per unit area of the individual layers were added together and related to the total cross-sectional area of the whole graft profile so as to allow for the calculation of the mean density of the nuclei profiles per whole sample. Two micrographs were taken for analysis purposes from each aorta, and in total 33,548 nuclei profiles were counted.

The relative position of the nuclei profiles within the graft was assessed using an arbitrarily defined function, *f*(*graft*), describing the relative distance of the profiles in the radial direction across the graft *f = d*1**/(*d*1* + d*2**), where *d*1 is the distance of the nuclei profiles from the outer (abluminal) graft border, and *d*2 is the distance of the nuclei profiles from the inner (adluminal, neointimal) graft border. The value *f* equaled 0 on the outer border and 1 on the inner border of the graft. In order to eliminate a potential edge effect [31] and the repeated counting of the nuclei profiles that crossed the borders and region of interest, the left adluminal part of each nucleus profile was deemed to be arbitrarily decisive concerning inclusion in or exclusion from the counting of the profile. It is important to note that this two-dimensional technique does not provide for the determination of the cell density unbiased by cell shape and size—this would require three-dimensional counting using a dissector volume probe in the thick sections of serial physical sections [32,33].

## 3. Results

### 3.1. Vascular Graft Morphology

**Figure 2:**
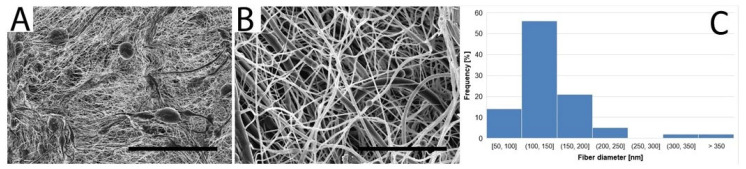
SEM images of a nanofibrous PCL vascular graft at lower (**A**) and higher (**B**) magnifications; scale bars 100 μm, 10 μm. Histogram of fiber diameter distribution (**C**).

The grafts were found to be composed mostly of thin fibers with a mean ± standard deviation of 183 ± 150 nm in diameter. The majority of fiber diameters was found to be between 100 and 150 nm, as seen in Figure 2C. Occasionally, thicker fibers having hundreds of nanometers in diameter were present within the structure. In addition to the fibers, polymeric beads were detected in the structures of the PCL grafts, as depicted in Figure 2A.

### 3.2. Vascular Graft Implantations and Patency

The PCL vascular grafts exhibited excellent surgical handling and suture retention properties during implantation. No significant blood leakage was observed following the restoration of blood flow and pressure. At both assessed time points, i.e., 10 days and 6 months, all the implanted grafts remained patent without exhibiting any signs of aneurysm formation.

### 3.3. Quantification of the Cell Distribution within the Vascular Graft

**Figure 3:**
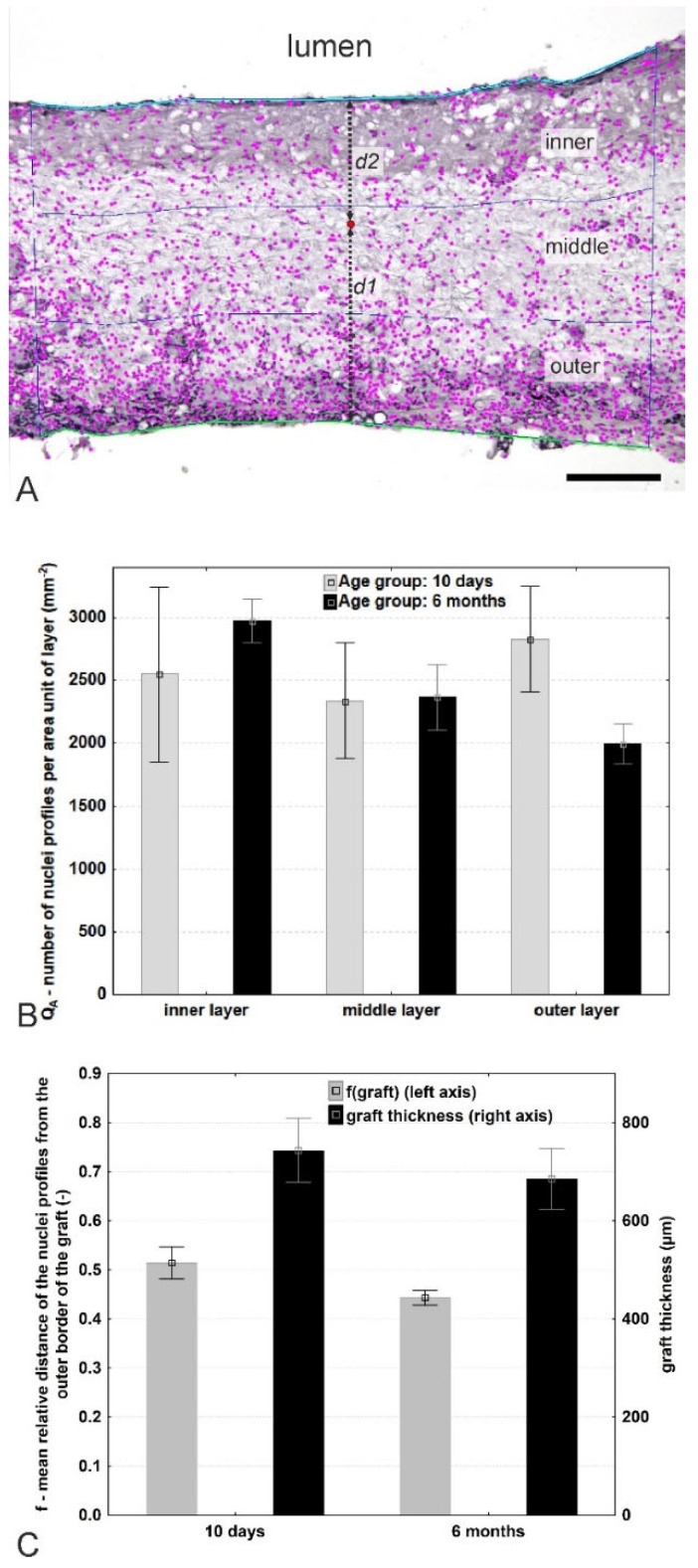
Hematoxylin-stained graft cross-section used for the evaluation of graft thickness and the relative distribution and density of the cell profiles infiltrating the grafts highlighting the inner and outer graft borders after 10 days of implantation (**A**). Quantification of cell nuclei per area unit of inner/middle/outer layer after 10 days (grey columns) and 6 months (black columns) (**B**). Graph showing mean relative distance of the nuclei profile from the outer border of the graft (left axis, grey columns), the graph representing graft thickness (black columns, right axis) (**C**). The data are presented as means ± standard error of the means (**B**,**C**).

A summary of the distribution of nuclei and their densities within the arbitrarily defined graft layers is provided in Figure 3. The graft thickness was automatically measured as the mean distance between both lines. Three virtual graft sublayers (inner, middle, and outer, each arbitrarily defined as comprising exactly 1/3 of the local thickness) were projected onto the micrograph. The nuclei profiles were semi-automatically mapped and their numbers and positions quantified in the corresponding sublayers. The relative position of the profiles within the graft was expressed as *f* = *d*1/(*d*1 + *d*2) (an example is provided at one point—marked in red in Figure 3A). After 10 days, a high degree of variability with respect to nuclei density was observed across the graft wall (Figure 3B, gray columns); however, after 6 months, a tendency was observed towards a cell density gradient that increased towards the inner layer (Figure 3B, black columns). With regard to the absolute numbers of cell nuclei, a small tendency was determined towards a shift in the mean relative distance of the cells to the middle layer after 6 months (Figure 3C, gray columns). Moreover, there was also a tendency towards decreased graft thickness after 6 months (Figure 3C, black columns). 

### 3.4. Endothelialization of the Graft Lumen

**Figure 4:**
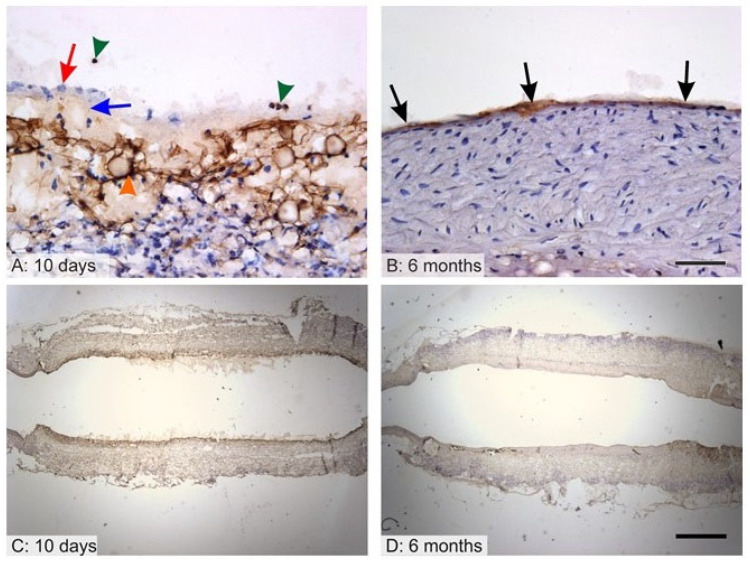
Endothelial coverage of the grafts after 10 days (**A**,**C**) and 6 months (**B**,**D**). Higher magnification photos show details of immunohistochemical staining; the samples are oriented with the lumen facing upwards (**A**,**B**). Photos with lower magnification depict the whole cross section of the graft under study (**C**,**D**). After 10 days, endothelial cells were present on peripheral regions of the grafts, and the middle of the graft exhibited no endothelial cell lining. The cells were negative for immunohistochemical staining but formed a monolayer (**A**—red arrow) on a thin acellular layer (**A**—blue arrow). Individual immune-positive circulating cells were found in the lumen (**A**—green arrowheads). The remnants of the graft showed a non-specific positive reaction (**A**—orange arrowhead). After 6 months (**B**,**D**), however, the grafts were seen to be completely covered with endothelium that was positive for the von Willebrand factor (**B**—black arrows). Scale bar 50 µm (**A**,**B**), 1000 µm (**C**,**D**).

The endothelialization of the graft lumen is considered to make up a key factor in terms of the patency of synthetic grafts since endothelial cells provide a constant non-thrombogenic surface. It was found that after 10 days, the proximal and distal parts adjacent to the aorta were lined with endothelium, leaving approximately the central half of the graft with no morphologically differentiated endothelium (Figure 4C). However, even the areas that lacked endothelium showed no signs of thrombosis. After 10 days, endothelium was negative or lacked consistent staining with concern to all the endothelial markers (von Willebrand factor, CD34, CD31) but remained morphologically distinct, forming a monolayer (Figure 4A, red arrow) that covered the adluminal surface and that lay on a thin acellular layer (Figure 4A, blue arrow). Accidentally, individual immuno-positive circulating cells were found in the lumen or adhering to the adluminal surface (Figure 4A, green arrowheads). The remnants of the grafts showed a non-specific positive reaction (Figure 4A, orange arrowhead). After 6 months, all the samples were completely covered with endothelium exhibiting positivity for the von Willebrand factor (Figure 4B,D) but negativity for the CD34 and CD31, leading to the assumption that the endothelial cells were in their unmatured state.

No intrinsic vascularization within the graft was observed after 10 days nor after 6 months of implantation. It was assumed that the graft allowed for the receiving of nutrition mainly from the lumen without any need for extensive *vasa vasorum* networks.

### 3.5. Vessel Remodeling

**Figure 5:**
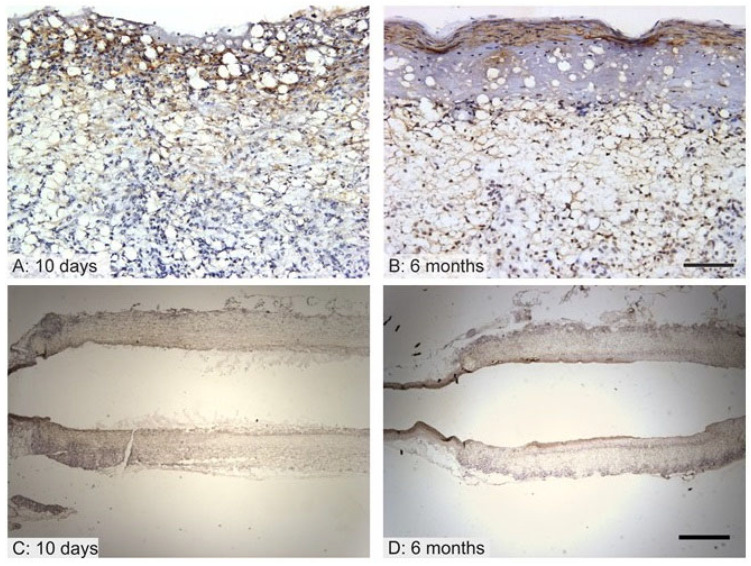
Immunohistochemical staining of alpha-smooth muscle actin (brown) after 10 days (**A**,**C**) and 6 months (**B**,**D**). Higher magnification photos show details of immunohistochemical staining; the samples are oriented with the lumen facing upwards (**A**,**B**). Photos with lower magnification depict the whole cross section of the graft under study (**C**,**D**). After 10 days, those cells that exhibited positivity for actin were found to be adjacent to the lumen of the PCL grafts (**A**). After 6 months, the grafts exhibited a well-differentiated vascular layer showing the regular arrangement of spindle-shaped smooth muscle cells (**B**). Scale bar 100 µm (**A**,**B**), 1000 µm (**C**,**D**).

**Figure 6:**
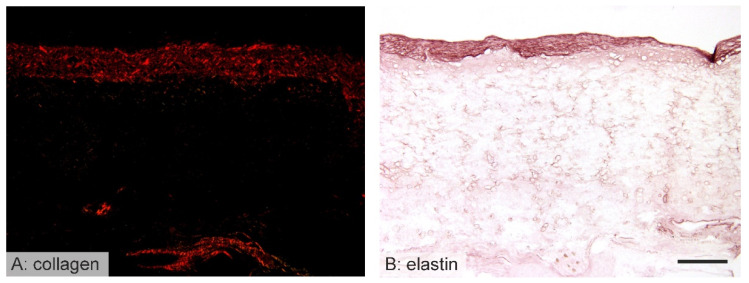
The collagen and elastin staining of the implanted grafts after 6 months. All the samples are oriented with the lumen facing upwards. Samples stained with picrosirius red (**A**) were used for the analysis of collagen deposition within the grafts. Samples stained with orcein were used for the assessment of the elastin fibers (dark brown) within the grafts (**B**). After 6 months, the grafts were covered with a neointimal endothelium, below which distinct elastic laminae had been deposited. Scale bars 200 µm.

Following the implantation of the vascular grafts, it was found that they were infiltrated by various types of cells. Smooth muscle cells and fibroblasts created an extracellular matrix, the purpose of which appeared to consist of the long-term replacement of the implanted vascular graft. Therefore, in order to investigate the hypothesis of the creation of structures resembling native blood vessels, the smooth muscle cells, collagen, and elastin depositions within the graft wall were stained. As expected, after 10 days there were few signs of such remodeling since the healing response was in its initial stages (smooth muscle cells had infiltrated the inner region of the implanted grafts). Six months following implantation, however, signs of regular aortic layers with concentric layers of elastin, vascular smooth muscle cells, and collagen were observed (Figure 5 and Figure 6).

### 3.6. Immunological Response to the Implanted Grafts

**Figure 7:**
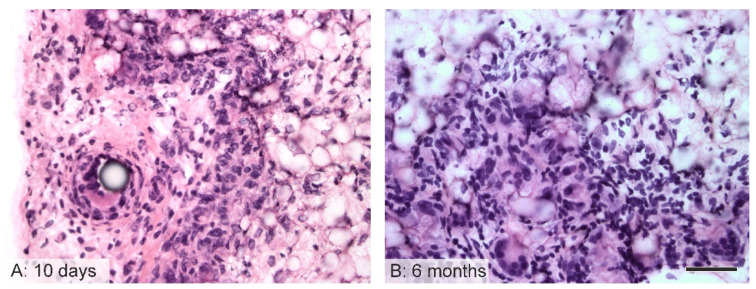
Multinucleated foreign-body giant cells (FBGC) in the grafts (hematoxylin–eosin staining). After 10 days, the FBGC were associated principally with the vascular stitches in a position adjacent to the aortic segments in the PCL grafts (**A**). After 6 months, FBGC were also frequently detected in the outer layers of the PCL grafts (**B**). Scale bar 50 µm.

The host inflammatory reaction was relatively mild with concern to all the samples. After 10 days, multinucleated foreign-body giant cells (FBGCs) were discovered associated principally with the stitches between the aortic wall and the grafts (Figure 7). After 6 months, foreign-body giant cells were also found close to the outer surfaces of the grafts.

### 3.7. Cartilaginous Metaplasia

**Figure 8:**
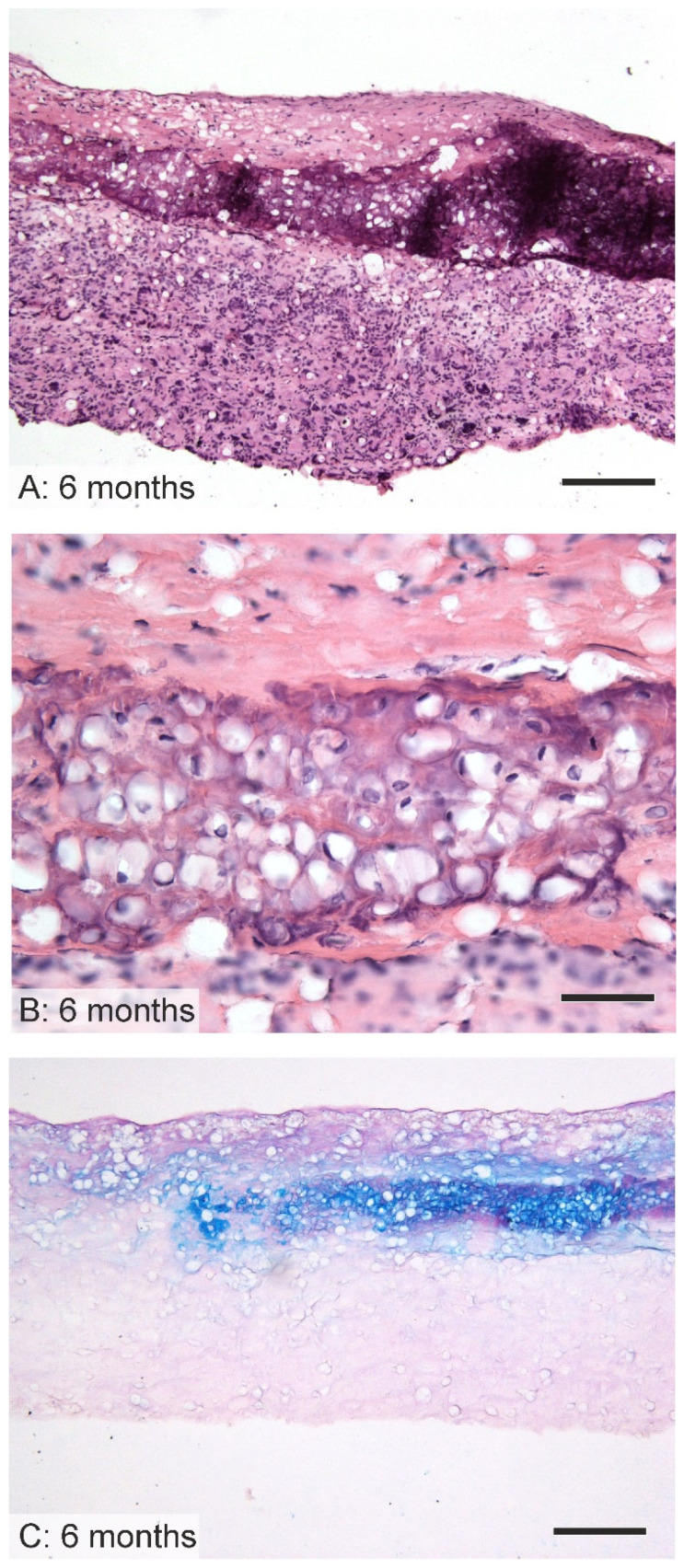
Cartilaginous metaplasia within the graft. After 6 months, one PCL graft contained areas of connective tissue morphologically resembling cartilage located in the vicinity of the junction between the graft and the aortic segments. The cells morphologically resembled chondroblasts and chondrocytes (**B**), and the extracellular matrix was weakly positive to neutral glycosaminoglycans (PAS staining, **A**) and strongly positive to acidic glycosaminoglycans (alcian blue staining, **C**) typical for the cartilaginous matrix. Hematoxylin–eosin (**A**,**B**); PAS combined with alcian blue (**C**); scale bar 200 µm (**A**,**C**), 50 µm (**B**).

Six months following implantation, signs of cartilaginous metaplasia were observed in a quarter of the PCL vascular grafts. The location of this transformation was determined as being in proximity to the stitches between the aorta junction and the graft (Figure 8).

## 4. Discussion

Small diameter single-layer biodegradable polycaprolactone vascular grafts with an inner diameter of 1.65 mm and a length of 2 cm were produced via the needle electrospinning technique for implantation purposes. The nanofibrous structure was composed of tiny fibers of around 180 nm in diameter with bead-like structures, as shown in Figure 2. While nanofibrous mats generally exhibit a high degree of porosity, small pore sizes may limit cellularization following implantation in vivo due to the high density of the compacted fibers. However, the resulting tubular prosthesis also contained beads that rendered the structure “fluffier”, thus allowing for enhanced cell infiltration. While other studies (listed in Table 1) addressed the evaluation of polycaprolactone with a higher molecular weight of 80,000 g/mol, our study aimed to prove the potential for the use of lower molecular weight polycaprolactone, i.e., 45,000 g/mol. Molecular weight influenced the material mechanical properties, degradation rate, etc. A comparison of polycaprolactone with lower (45,000) and higher (80,000) molecular weight was conducted in our previous study [12]. It was found that polycaprolactone with lower molecular weight possessed an ultimate tensile strength of 0.15 MPa and an elongation at break of 74% compared to higher molecular weight polycaprolactone, which had a tensile strength of 2.04 MPa and an elongation at break of 150%. However, this study compared the graft thickness of 200 µm. In this study, wall thickness was adjusted to 700 µm (as can be seen in Figure 3C) in order to ensure sufficient mechanical properties of implanted grafts. The vascular grafts were implanted in rats as a replacement for the abdominal aorta. The healing response of the prostheses was analyzed 10 days and 6 months following implantation in order to assess the short-term outcomes and long-term response. The patency rate of the implanted prostheses was found to have reached 100% at both the assessed time points. In order to evaluate the regeneration potential of the biomaterial, a complete histological assessment was conducted. Longitudinal cross-sections of the graft with neighboring tissue were obtained in order to analyze the changes that had occurred within the whole length of the prosthesis. While such an approach is relatively complicated in terms of processing, the outcomes are of particular value with respect to the assessment of all the parts of the graft (the middle section as well as the anastomoses between the graft and the aortic wall). Immunohistochemical rather than immunofluorescence staining was preferred due to the autofluorescence of polycaprolactone, indicating false positivity.

Stereological assessment provides objective unbiased quantification of specific parameters. Stereological approaches were utilized in tissue engineering of bone [13,14], cartilage [34], nerve [35], etc. In the field of cardiovascular tissue engineering, stereological tools (point counting) were used in study of Van Vré et al. where cross sections of natural blood vessel areas (lumen, *tunica intima*, *tunica media*) were quantified by the stereological method compared to planimetry [36].

However, the regeneration of vascular graft has never been evaluated by stereological methods based on our best knowledge. Wang et al. analyzed the cellularization of a vessel wall in distinct layers of 100 μm thickness [21]. Since the thickness of the prosthesis may change during the course of the regeneration process, we decided on a universal approach, according to which the cell distribution within the graft was evaluated by means of a new technique. The graft thickness was divided into three distinct parts, in each of which the cell distribution was assessed. The native blood vessel is composed of three distinct layers that differ in terms of thickness and composition, namely, the *tunica intima*, *tunica media*, and *tunica adventitia*, and the implantation of the polymeric prosthesis was conducted so as to lead to the regeneration of these three layers in vivo. It was decided to distinguish between the adluminal (inner), middle, and abluminal (outer) parts in order to be able to quantify the regeneration process in different parts of the prosthesis. At the earlier investigated time point of 10 days, the grafts had been infiltrated homogeneously throughout the graft thickness with around 2500 cell nuclei profiles per mm^2^ (inner layer 2550 ± 1201, middle layer 2340 ± 803, outer layer 2829 ± 729), which was explained by the rapid cellularization of the graft by immune cells that were able to penetrate into the whole graft thickness of 744 ± 56 μm due to the high degree of porosity of the nanofibrous structure. No preference with respect to outer or inner site cell origin was detected. After 6 months of healing, the cell distribution had changed, i.e., the inner layer was seen to have become more densely cellularized (2976 ± 353 cell nuclei profiles per mm^2^). Moreover, the cell number was observed to have decreased with distance from the lumen of the graft (the middle layer contained 2367 ± 523 cells per mm^2^, the outer layer 1997 ± 311). The regeneration of the graft wall was observed to progress from the inner site as evinced by the specific staining, as discussed further. The thickness of the graft had decreased slightly after 6 months to 686 ± 62 μm, which could be explained by the partial degradation of the polymer. Penetrated cells, especially immune cells, produce enzymes that may act to quicken the degradation rate of polycaprolactone: firstly, the PCL is degraded via the non-enzymatic hydrolytic cleavage of ester bonds, which leads to increased crystallinity and a decrease in the molecular weight of the polymer, followed by the enzymatic degradation of the PCL in the macrophages [6].

The endothelization of the graft lumen was observed after 6 months. Endothelial cells proliferated through the anastomosis as depicted in the assessment of the early stage (see Figure 4). Since the early endothelial cells did not express their natural markers such as CD 31 and CD 34, it was assumed that they were not completely differentiated. However, negativity for CD31 does not necessarily demonstrate the premature state of the endothelium, as confirmed by other studies [37,38]. It is possible that the nanofibrous structure itself hastened endothelial cell proliferation, as stated in the study by Pektok et al., which compared nanofibrous PCL to inert ePTFE grafts [5]. The complete endothelialization of the PCL graft lumen after 6 months was also confirmed by de Valence et al. and was observed to remain stable for up to 18 months in rats [7]. Surprisingly, no vascularization was observed in any of the samples; it is probable that the whole thickness of the graft was nourished by diffusion from the vessel lumen and that there was no need for external vascularization. Other studies observed the formation of *vasa vasorum* in electrospun PCL grafts in the first months following implantation with further regression [7,23].

The implantation of biodegradable polymers serves to provide a temporary scaffold that is replaced by an extracellular matrix (ECM) of the final tissue during the remodeling phase. The process commences with the infiltration of the scaffold by host cells, which is followed by the synthesis of a typical tissue ECM. In the case of the implanted vascular grafts, smooth muscle cells penetrated the graft and synthesized the collagen and elastin fibers in the same way as in native vessels, as can be seen in Figure 5 and Figure 6. The PCL grafts created individual islands of smooth muscle cells that produced collagen and elastin for the remodeled vessel. Similar results have also been determined for higher molecular weight polycaprolactone-based implants [8,9,21,22,23].

The immunological response to the implanted biomaterial was as anticipated. The grafts were observed not to be surrounded by a collagen capsule that might potentially have limited the healing response and the remodeling phase. Only a small number of foreign body giant cells were found in the vicinity of the stitches and the outer part of the implanted nanofibrous grafts. Since polycaprolactone is a biodegradable polymer, it was assumed that the rat immune system cooperated with the material and “allowed” the initiation of the healing and remodeling phase, as suggested by the specific staining of the cells and the ECM.

After 6 months in vivo, one of implanted grafts showed signs of cartilaginous metaplasia, as demonstrated in Figure 8; a similar observation was noted by de Valence et al. and Li et al. [7,23]. We discovered an island of cells that was typical of cartilaginous tissue, the reason for which may have been the mismatch between the thicknesses of the rat adjacent aorta and the implanted grafts, i.e., the grafts were designed to be significantly thicker than the native aorta in order to ensure the required mechanical properties of the final prostheses. In this case, the grafts may have been exposed to differing mechanical stimuli, thus resulting in the observed cartilaginous metaplasia. The lower molecular weight of the PCL used in the study did not prevent the initiation of the calcification process.

The overall assessment of a nanofibrous-based polycaprolactone vascular graft provided herein indicates that such a material is able to regenerate the native vessel wall in rats, as can be seen in Figure 4, Figure 5 and Figure 6. On the other hand, adverse effects such as foreign body reaction and cartilaginous metaplasia occurred (Figure 7 and Figure 8). Similar outcomes were described also in studies based on higher molecular weight PCL [7,23]. Therefore, molecular weight probably does not play an important role in the regeneration of the vessel wall. While the initial implantation of low molecular weight polycaprolactone was investigated as proof of the concept of the material, the low number of investigated samples and, thus, the inability to conduct a statistical comparison formed one of the main limitations of the study. Further work is planned involving the comparison of electrospun PCL vascular grafts with higher molecular weights using higher animal models and applying a similar histological evaluation approach. Moreover, it will be necessary to conduct a detailed assessment of the short- as well as the long-term results. In order to prevent the occurrence of adverse effects such as cartilaginous metaplasia, the testing is planned of the modification of the material used in the study.

## 5. Conclusions

The study provides new insight into the vessel remodeling process and the assessment of nanofibrous low molecular weight polycaprolactone vascular grafts. The grafts remained patent and mechanically stable for 6 months following implantation. Cellularization of graft was assessed by a novel stereological approach. It was found that the graft wall had become homogeneously penetrated by cells after 10 days in vivo. The regeneration of the vessel wall was observed at the inner site where the majority of cells was present after 6 months. The prosthesis lumen was endothelialized after 6 months without the detection of *vasa vasorum* within the graft. Concentric layers of smooth muscle cells producing collagen and elastin were found under the endothelium, resembling the native vessel wall architecture. No severe immunological reaction was detected around the implanted biomaterial. The adverse effect of cartilaginous metaplasia close to the anastomoses was detected in one of the implanted grafts (25%).

## Figures and Tables

**Table 2:** Histological staining methods and their purpose.

Staining	Purpose
hematoxylin–eosin [24]	overall morphology of the graft, foreign-body giant cells
hematoxylin only	nuclei of the cells infiltrating the graft
Verhoeff’s hematoxylin and green trichrome [25]	overall morphology, differentiation of the connective tissue, elastin and vascular smooth muscle
orcein (Tanzer’s orcein, Bowley Biochemical Inc., Danvers, MA, USA)	elastin fibers
picrosirius red (Direct Red 80, Sigma Aldrich, Munich, Germany)	type I and type III collagen when observed under circularly polarized light
PAS and alcian blue [26,27]	additional staining of the samples with cartilaginous metaplasia: PAS demonstrated neutral hexoses or sialic acid, alcian blue binds to acidic glycosaminoglycans at a pH of 2.5

**Table 3:** Primary antibodies used with respect to immunohistochemistry.

Antibody (and Staining Purpose)	Manufacturer	Dilution	Pretreatment
Monoclonal Mouse Anti-Human Smooth Muscle Actin, Clone 1A4 (*marker of smooth muscle and myofibroblasts*)	DakoCytomation (Glostrup, Denmark)	1:100	20 min 96 °C Dako Target Retrieval Solution, pH 6
Monoclonal Anti-CD34 antibody (*endothelial marker*)	Abcam (Cambridge, MA, USA)	1:250	20 min 96 °C Dako Target Retrieval Solution, pH 6
Monoclonal Anti-CD31 antibody Clone J70A (*endothelial marker*)	DakoCytomation)	1:40	20 min 96 °C Dako Target Retrieval Solution, pH 9
Polyclonal Rabbit, Anti-Human, von Willebrand factor, Code A0082 (*endothelial marker*)	DakoCytomation	1:1000	10 min in chilled acetone, Proteinase K

**Table 4:** Quantitative parameters analyzed in the vascular grafts (2 images from the central part of the graft, objective 10×).

Quantitative Parameter Abbreviation	Definition, Reference Space, Interpretation, and Units
Q_A_-inner layer	Number (or two-dimensional density) of nuclei profiles found within the innermost (adluminal) third of the graft thickness in a transverse section (mm^−2^).
Q_A_-middle layer	Number (or two-dimensional density) of nuclei profiles found within the middle third of the graft thickness in a transverse section (mm^−2^).
Q_A_-outer layer	Number (or two-dimensional density) of nuclei profiles found within the outer (abluminal) third of the graft thickness in a transverse section (mm^−2^).
Q_A_-mean per graft	The mean number (or two-dimensional density) of nuclei profiles found within the whole graft in a transverse section (mm^−2^).
f(graft)	Mean relative distance of the nuclei profiles found within the graft from the outer border of the graft. A dimensionless parameter ranging between 0 and 1, where the value = 0 refers to the nuclei profiles directly on the outer graft border, and the value = 1 refers to the nuclei on the luminal border of the neointima (-).
Graft thickness	Thickness of the graft measured as the mean distance between the adluminal (neointimal) surface profile (line 1) and the outer abluminal (outer) border profile (line 2) (µm).

## Data Availability

Not applicable.
